# New contributions to the molecular systematics and the evolution of host-plant associations in the genus *Chrysolina* (Coleoptera, Chrysomelidae, Chrysomelinae)

**DOI:** 10.3897/zookeys.547.6018

**Published:** 2015-12-17

**Authors:** José A. Jurado-Rivera, Eduard Petitpierre

**Affiliations:** 1 Departament de Biologia, Universitat de les Illes Balears, 07122 Palma de Mallorca, Spain; 2 Institut Mediterrani d’Estudis Avançats, CSIC, Miquel Marquès 21, 07190 Esporles, Balearic Islands, Spain

**Keywords:** Coleoptera, Chrysomelidae, *Chrysolina*, *Oreina*, Phylogeny, Insect-plant interaction, *cox*1, *rrnL*, H3

## Abstract

The taxonomic circumscription of the large and diverse leaf beetle genus *Chrysolina* Motschulsky is not clear, and its discrimination from the closely related genus *Oreina* Chevrolat has classically been controversial. In addition, the subgeneric arrangement of the species is unstable, and proposals segregating *Chrysolina* species into new genera have been recently suggested. In this context, the availability of a phylogenetic framework would provide the basis for a stable taxonomic system, but the existing phylogenies are based on few taxa and have low resolution. In the present study we perform a phylogenetic analysis based on mitochondrial (*cox*1 and *rrnL*) and nuclear (H3) DNA sequences from a sample of fifty-two *Chrysolina* species representing almost half of the subgeneric diversity of the group (thirty out of sixty-five subgenera) and most of the morphological, ecological and karyological variation in the genus. In addition, five *Oreina* species from two subgenera have also been analysed. The resulting phylogeny is used to evaluate some of the most relevant taxonomic hypotheses for *Chrysolina*, and also to reconstruct its ancestral host plant associations in a Bayesian framework. Our findings support the paraphyly of *Chrysolina* as currently defined due to the inclusion of *Oreina*, the monophyly of the *Chrysolina* (plus *Oreina*) species including the divergent Chrysolina (Polysticta) vigintimaculata (Clark, 1864), and enable inferences of deep-level evolutionary relationships among the studied subgenera. The plant family Lamiaceae is inferred as the ancestral host of the study group, whose evolution is characterized by continuous host-shifting among pre-existing host plant families. Some *Chrysolina* clades include mixtures of species with different levels of diet breadth, indicating that niche width has varied through time.

## Introduction

The genus *Chrysolina* Motschulsky is a very large and diverse group of leaf-beetles that are mainly distributed in Europe, Asia and Africa (Bieńkowski 2001). Nearly 450 species belonging in 65 subgenera have been recognized (Bieńkowski 2001), and new species are still being described (e.g. Ge et al. 2011, Bourdonné et al. 2013, Lopatin 2011, 2014). However, despite the number of taxonomic studies focused on *Chrysolina* its taxonomy is chronically confused (Kippenberg 2010), and even the circumscription of the genus remains unclear. In fact, the most recent and updated taxonomic review (Bieńkowski 2007) does not contribute a comparative morphological diagnosis to differentiate *Chrysolina* from the closely related genera. In this sense the most controversial case is the one concerning the genera *Chrysolina* and *Oreina* Chevrolat, whose discrimination mainly relies in the ratio between the length of the metasternum and the length of the first abdominal sternite (Weise 1893). It has been suggested that this morphological attribute could be inconsistent (Bieńkowski 2007), thus reinforcing the inclusion of the genus *Oreina* within *Chrysolina* (Chapuis 1874, Bourdonné and Doguet 1991, Daccordi 1994) or conversely the recognition of *Chrysolina* as a subgenus of *Oreina* (Monrós and Bechyné 1956). In addition, taxonomic rearrangements are frequent in *Chrysolina*, including decisions splitting species into new genera (e.g. *Craspeda* Motschulsky [=*Zeugotaenia* Motschulsky]: Bourdonné 2005, *Camerounia* Jolivet: Bieńkowski 2007, *Chalcoidea* Motschulsky: Bourdonné 2012). Likewise, the subgeneric arrangement of the *Chrysolina* species is also unstable (Mikhailov 2000, 2002, Bieńkowski 2001, 2007, Bourdonné 2008, 2012, Kippenberg 2010). This taxonomic instability reflects the lack of a supraspecific systematic for the genus *Chrysolina*, due in part to the absence of a phylogenetic background.

Phylogenetic studies focused on *Chrysolina* are scarce and limited to a reduced number of taxa. Bourdonné and Doguet (1991) proposed the first evolutionary hypothesis for 10 groups of Palaearctic species attending to both their chromosome numbers and host-plant affiliations. From a molecular perspective, Garin et al. (1999) performed a phylogenetic analysis based on mtDNA sequences (*cox*1 and *rrnL*) from 30 *Chrysolina* species representing 22 subgenera plus two *Oreina* species. The resulting phylogenetic trees allowed for the identification of monophyletic lineages comprising few species each, but the deep level relationships were poorly resolved. On the other hand, the two *Oreina* species nested within the *Chrysolina* clade, but this relationship was unsupported. Simultaneously, Hsiao and Pasteels (1999) also inferred a molecular phylogeny based on mtDNA markers (12S and *rrnL*) from 16 *Chrysolina* species ascribed to 14 subgenera and 14 *Oreina* species, but the resulting topologies also had low resolution at the basal nodes. *Oreina* species were recovered as a monophyletic lineage that also included *Chrysolina fastuosa* (Scopoli, 1763), and all of them were nested in the *Chrysolina* clade. Both molecular studies highlighted the reciprocal monophyly of the subgenera *Melasomoptera* Bechyné and *Synerga* Weise, and of *Hypericia* Bedel and *Sphaeromela* Bedel, however discrepancies were observed regarding the systematic position of the subgenera *Colaphodes* Motschulsky and *Taeniochrysea* Bechyné.

Apart from taxonomic purposes, the availability of a phylogenetic hypothesis for the species of *Chrysolina* may allow for the study of evolutionary processes such as their ancestral host plant affiliations. In this regard, this leaf beetle genus constitutes a suitable and interesting study group as most of the species are oligophagous, each of them feeding on a narrow range of closely related plants (Jolivet and Petitpierre 1976, Bourdonné and Doguet 1991). Indeed, the taxonomic conservatism in host plant use found in *Chrysolina* is so high that host use has been frequently coupled with other systematic characters to circumscribe species assemblages (Petitpierre and Segarra 1985, Bourdonné and Doguet 1991, Petitpierre and Mikhailov 2009). The ancestral reconstruction of the trophic affiliations in *Chrysolina* and *Oreina* was addressed in the phylogenetic studies performed by Garin et al. (1999) and by Hsiao and Pasteels (1999), inferring the plant family Lamiaceae as the most likely ancestral host for *Chrysolina* + *Oreina* (Garin et al. 1999) and the Asteraceae for *Oreina* (Hsiao and Pasteels 1999). However, these reconstructions were based on poorly resolved phylogenetic trees from few taxa.

In this work we present the results of a phylogenetic study based on mitochondrial and nuclear DNA sequences from a sample of *Chrysolina* and *Oreina* species, using Bayesian and maximum likelihood (ML) inference approaches. We expand the taxon sampling of previous molecular studies (Garin et al. 1999, Hsiao and Pasteels 1999) through the inclusion of representatives for nearly half of the *Chrysolina* subgenera comprising most of the morphologically defined groups and ecological variation of the genus. In addition, the inferred molecular phylogeny is used to test the validity of a number of taxonomic hypotheses derived from morphological, ecological, chemical and genetic data. Finally, we aim to investigate the evolution of the host plant associations in the genus *Chrysolina*.

## Materials and methods

### Taxon sampling

We have studied 52 *Chrysolina* species representing 30 out of the *ca.* 65 subgenera currently recognized for the genus (Bieńkowski 2001, Kippenberg 2010), plus five *Oreina* species from two subgenera. Our sampling includes type species representatives regarding 13 of the studied *Chrysolina* subgenera and one type species for *Oreina*. In addition, several representatives of other genera of the subfamily Chrysomelinae were analysed as outgroups, including a species from the early-divergent genus *Timarcha* Latreille (Gómez-Zurita et al. 2008) (Table 1). Beetles were collected by us in the field or received from colleagues in absolute ethanol and stored in the laboratory at -20 °C before processing. Voucher specimens are deposited for long-term storage at the DNA and tissue collection of the Biodiversity, Systematics and Evolution group (Bio6Evo) of the University of the Balearic Islands.

**Table 1.** Studied taxa, sources, host plants and GenBank accession numbers. Species groups defined by Bourdonné and Doguet (1991) are also indicated. a: Baselga and Novoa (2006), b: Bieńkowski 2010, c: Bieńkowski 2011, d: Bourdonné 2005, e: Bourdonné and Doguet 1991, f: Cobos 1953, g: Garin et al. 1999, h: Jolivet and Petitpierre 1976, i: Jolivet et al. 1986, j: Koch 1992, k: Lopatin and Mikhailov 2010, l: Mikhailov 2006, m: Petitpierre 1981, n: Rizza and Pecora 1980, o: Vela and Bastazo 1999.

Species	Source	Host(s)	Host(s) references	Bourdonné and Doguet’s (1991) group	cox1	rrnL	H3
*Chrysolina aeruginosa* (Faldermann, 1835)	SE Tuva, Siberia, Russia	Asteraceae (*Artemisia*), Lamiaceae (*Thymus*)	b		LN833682	LN833808	LN833745
*Chrysolina baetica* (Suffrian, 1851)	Murcia, Spain	Lamiaceae (*Satureja*, *Thymus*)	i	2	LN833683	LN833809	LN833746
*Chrysolina americana* (Linnaeus, 1758)	Almuñecar, Spain	Lamiaceae (*Lavandula*, *Rosmarinus*)	b, h	2	LN833684	LN833810	LN833747
*Chrysolina aurichalcea* (Gebler in Mannerheim, 1825)	Ticino, Switzerland	Apocynaceae (*Vincetoxicum officinale*), Asteraceae (*Arctium*, *Artemisia*, *Aster*, *Kalimerus*, *Petasites*)	b, j	9	LN833685	LN833811	LN833748
*Chrysolina banksi* (Fabricius, 1775)	Balearic Islands, Spain	Lamiaceae, Plantaginaceae	h	2	LN833686	LN833812	LN833749
*Chrysolina bicolor* (Fabricius, 1775)	Canary Islands, Spain	Lamiaceae (*Saccocalyx*, *Salvia*, *Thymus*)	h	2	LN833687	LN833813	LN833750
*Chrysolina carnifex* (Fabricius, 1792)	Barcelona, Spain	Asteraceae (*Artemisia*, *Santolina*)	b	9	LN833688	LN833814	LN833751
*Chrysolina cerealis cyaneoaurata* (Motschulsky, 1860)	Altai, Siberia, Russia			2	LN833689	LN833815	LN833752
*Chrysolina colasi* (Cobos, 1952)	Granada, Spain	Lamiaceae (*Sideritis glacialis*)	o	1	LN833690	LN833816	LN833753
*Chrysolina convexicollis* (Jakobson, 1901)	SE Tuva, Siberia, Russia	Asteraceae (*Artemisia*)	c		LN833691	LN833817	LN833754
*Chrysolina costalis* (Olivier, 1807) (=*Chrysolina obsoleta* Brullé, 1838 *sensu* Bieńkowski 2014 unpubl.)	Canary Islands, Spain	Ranunculaceae (*Ranunculus*)	e	2	LN833714	LN833818	LN833777
*Chrysolina diluta* (Germar, 1824)	Granada, Spain	Plantaginaceae (*Plantago*)	h	3	LN833693	LN833819	LN833756
*Chrysolina eurina* (Frivaldszky, 1883: 17)	Mundybash, Kemerovskaya oblast’, Russia	Asteraceae (*Tanacetum vulgare*)	b	9	LN833694	LN833820	LN833757
*Chrysolina fastuosa* (Scopoli, 1763)	Lleida, Spain	Lamiaceae (*Galeopsis*, *Lamium*, *Leonorus*, *Prunella*)	h, i	2	LN833695	LN833821	LN833758
*Chrysolina femoralis* (Olivier, 1790)	Girona, Spain	Lamiaceae (*Satureja*, *Thymus*)	h, i	2	LN833696	LN833822	LN833759
*Chrysolina fuliginosa* (Olivier, 1807)	Lleida, Spain	Asteraceae (*Centaurea*)	h	9	LN833697	LN833823	LN833760
*Chrysolina gemina* (Brullé, 1838)	Canary Islands, Spain	Lamiaceae (*Lavandula*)	h	2	LN833698	LN833824	LN833761
*Chrysolina geminata* (Paykull, 1799)	Lleida, Spain	Hypericaceae (*Hypericum*)	b	10	LN833699	LN833825	LN833762
*Chrysolina haemochlora* (Gebler, 1823)	Ust’-Koksa, Altai Republic, Russia	Apiaceae (*Aegopodium*, *Angelica*, *Conioselinum*, *Heracleum*, *Pleurospermum*)	c		LN833700	LN833826	LN833763
*Chrysolina haemoptera* (Linnaeus, 1758)	La Coruña, Spain	Plantaginaceae (*Plantago*)	m	7	LN833701	LN833827	LN833764
*Chrysolina helopioides* (Suffrian, 1851)	Málaga, Spain	Apiaceae (*Ferula*)	h	4	LN833702	LN833828	LN833765
*Chrysolina herbacea* (Duftschmid, 1825)	Teruel, Spain	Lamiaceae (*Mentha*)	b, h	2	LN833703	LN833829	LN833766
*Chrysolina hyperici* (Forster, 1771)	Bragança, Portugal	Hypericaceae (*Hypericum*)	b	10	LN833704	LN833830	LN833767
*Chrysolina jakowlewi* (Weise, 1894)	Sayan Mts., Tuva, Russia				LN833705	LN833831	LN833768
*Chrysolina janbechynei* Cobos, 1953 [= *Chrysolina curvilinea* (Weise, 1884)]	Murcia, Spain	Asteraceae (*Artemisia*)	f	9	LN833692	LN833832	LN833755
*Chrysolina kocheri* (Codina Padilla, 1961)	Smimou, Morocco	Plantaginaceae (*Plantago coronopus*)	d	3	LN833706	LN833833	LN833769
*Chrysolina kuesteri* (Helliesen, 1912)	Tejeda, Granada, Spain	Lamiaceae, Scrophulariaceae (*Linaria*)	b, e	1	LN833707	LN833834	LN833770
*Chrysolina lepida* (Olivier, 1807)	Huéscar, Granada, Spain	Asteraceae (*Mantisalca salmantica*)	e	9	LN833708	LN833835	LN833771
*Chrysolina lucida* (Olivier, 1807)	Almería, Spain	Lamiaceae (*Mentha*)	h	2	LN833709	LN833836	LN833772
*Chrysolina lucidicollis grossepunctata* (Lindberg, 1950)	Canary Islands, Spain	Scrophulariaceae (*Linaria*)	e	1	LN833710	LN833837	LN833773
*Chrysolina marginata* (Linnaeus, 1758)	Girona, Spain	Asteraceae (*Achillea*)	b, e, h	9	LN833711	LN833838	LN833774
*Chrysolina affinis mesatlantica* (Kocher, 1958)	Moyen Atlas, Morocco			2	LN833712	LN833839	LN833775
*Chrysolina obscurella* (Suffrian, 1851)	Var, France	Apiaceae	e	4	LN833713	LN833840	LN833776
*Chrysolina oirota* Lopatin, 1990	Ivanovsky massif, Kazakhstan	Asteraceae (*Saussurea latifolia*), Lamiaceae (*Lamium*)	k		LN833715	LN833841	LN833778
*Chrysolina pedestris* (Gebler, 1823)	Sekisovka, Kazakhstan	Apiaceae (*Seselis*)	c		LN833716	n.a.	LN833779
*Chrysolina peregrina* (Herrich-Schaeffer, 1839)	Balearic Islands, Spain	Apiaceae (*Daucus*, *Phoeniculum*)	g, h	8	LN833717	n.a.	LN833780
*Chrysolina perforata* (Gebler, 1830)	Erzin, Russia	Asteraceae, Lamiaceae	c		LN833718	LN833842	LN833781
*Chrysolina petitpierrei* Kippenberg, 2004	Lleida, Spain				LN833719	LN833843	LN833782
*Chrysolina polita* (Linnaeus, 1758)	Girona, Spain	Lamiaceae (*Lycopus*, *Mentha*, *Origanum*, *Satureja*)	b, h, i	2	LN833720	LN833844	LN833783
*Chrysolina quadrigemina* (Suffrian, 1851)	Bragança, Portugal	Hypericaceae (*Hypericum*)	h	10	LN833721	LN833845	LN833784
*Chrysolina reitteri* (Weise, 1884)	Susuz, Turkey				LN833722	LN833846	LN833785
*Chrysomela rossia* (Illiger, 1802)	Torino, Italy	Lamiaceae (*Mentha piperita*), Scrophulariaceae (*Linaria*, *Veronica*)	b, n	1	LN833723	LN833847	LN833786
*Chrysolina rufoaenea* (Suffrian, 1851)	Zamora, Spain	Apiaceae (*Carum verticillatum*)	a, i	8	LN833724	LN833848	LN833787
*Chrysolina soiota* (Jakobson, 1924)	Kulumys range, Oisky pass, Russia				LN833726	LN833849	LN833789
*Chrysolina sturmi* (Westhoff, 1882)	Chelyabinsk, Russia	Asteraceae (*Cirsium*), Lamiaceae (*Glechoma*), Scrophulariaceae (*Linaria*)	b		LN833727	n.a.	LN833790
*Chrysolina sylvatica* (Gebler, 1823)	Kulumys range, Oisky pass, Russia	Ranunculaceae (*Aquilegia glandulosa*)	l		LN833728	LN833850	LN833791
*Chrysolina timarchoides* (Brisout de Barneville, 1882)	Girona, Spain	Apiaceae (*Bupleurum*, *Heracleum*)	h	4	LN833729	LN833851	LN833792
*Chrysolina tundralis* (Jakobson, 1910)	Serebryansky Mount, Russia	Asteraceae (*Arnica*, *Saussurea*), Lamiaceae (*Lamium purpureum*)	c		LN833730	LN833852	LN833793
*Chrysolina vernalis pyrenaica* (Dufour, 1843)	Lleida, Spain	Plantaginaceae (*Plantago*)	m	7	LN833731	LN833853	LN833794
*Chrysolina vigintimaculata* (Clark, 1864)	KwaZulu-Natal, South Africa				LN833732	n.a.	LN833795
*Chrysolina viridana* (Kuster, 1844)	Riofrio, Granada, Spain	Lamiaceae (*Mentha*)	h	2	LN833733	LN833854	LN833796
*Chrysolina wollastoni* (Bechyné, 1957) [=*Chrysolina rutilans* (Wollaston, 1864)]	Canary Islands, Spain	Lamiaceae (*Mentha*)	h	2	LN833725	LN833855	LN833788
*Oreina cacaliae* (Schrank, 1785)	Lleida, Spain	Asteraceae (*Adenostyles*, *Petasites*)	i	6	LN833735	LN833857	LN833798
*Oreina fairmairiana* (De Gozis, 1882) [=*Oreina splendidula* (Fairmaire, 1865)]	Lleida, Spain	Apiaceae, Asteraceae (*Senecio*)	e	6	LN833739	LN833858	LN833802
*Oreina ganglbaueri* (Jakob, 1953)	Lleida, Spain	Apiaceae (*Angelica*, *Heracleum*, *Meum*)	i	5	LN833736	LN833859	LN833799
*Oreina speciosa* (Linnaeus, 1767)	Massif des Vosges, Haut-Rhin, France	Apiaceae (*Angelica*, *Heliosiadium*, *Laserpitium*, *Peucedanum*)	i	5	LN833737	n.a.	LN833800
*Oreina speciosissima* (Scopoli, 1763)	Lleida, Spain	Asteraceae (*Adenostyles*, *Cirsinus*, *Petasites*, *Senecio*)	i	6	LN833738	LN833860	LN833801
*Lamprolina aeneipennis* (Boisduval, 1835)	Mount Keira, NSW, Australia				LN833734	LN833856	LN833797
*Paropsis atomaria* Olivier, 1807	Molonglo Gorge Nature Reserve, ACT, Australia				LN833740	LN833862	LN833803
*Paropsisterna liturata* (Marsham, 1808)	Black Mountain, ACT, Australia				LN833741	LN833861	LN833804
*Phyllocharis cyanicornis* (Fabricius, 1801)	Royal National Park, NSW, Australia				LN833742	LN833863	LN833805
*Poropteromela epipleuralis* Lea, 1916	Mount Moombil, NSW, Australia				LN833743	LN833864	LN833806
*Timarcha sinuatocollis* Fairmaire 1861	Lleida, Spain				LN833744	LN833865	LN833807

### DNA isolation, PCR amplification and sequencing

Total DNA was purified from beetle head and pronotum using the DNeasy Tissue kit (Qiagen, West Sussex, UK) and following the manufacturer’s protocol. Elutions were done in 200 μL volume and one microliter was used in PCR reactions. Three different molecular markers were selected for the study, including a partial sequence of the mitochondrial 16S rDNA (*rrnL*; primers LR-N-13398 and LR-J-12887; Simon et al. 1994), a partial sequence of the mitochondrial cytochrome c oxidase subunit 1 gene (*cox*1; primers C1-J-2183 and TL2-N-3014; Simon et al. 1994), and a fragment from the nuclear histone 3 gene (H3; primers H3aF and H3aR; Colgan et al. 1998). PCR conditions used 0.2 μM of each primer and 3.5 mM MgCl_2_ using a standard protocol of 35 cycles with annealing temperature ranging from 50 to 45 °C (60s) depending on the sample, and denaturation (94 °C) and elongation (72 °C) lasted 30 and 60s, respectively. PCR products were visualized by 1% agarose gel electrophoresis and subsequently purified using MSB Spin PCRapace (Invitek, Berlin, Germany). Sanger sequencing was performed with the same primers as above using the BigDye Terminator Cycle Sequencing kit (Applied Biosystems, Foster City, CA, USA). Sequences were edited and contigs were assembled using BIOEDIT v. 7 (Hall 1999), and deposited at GenBank under the accession numbers referred in Table 1.

### Phylogenetic analyses

Heterogeneity in base composition across taxa was explored for each codon position of the protein-coding genes and for *rrnL* using the chi-square test for base frequency differences implemented in PAUP*4.0b10 (Swofford 2003). Multiple sequence alignment was performed using MAFFT 7 online version (http://mafft.cbrc.jp/alignment/server/, Katoh and Standley 2013) under default parameters. Molecular markers were checked for combinability using the incongruence length difference (ILD) test (Farris et al. 1994) implemented in PAUP* v4.0b10 (Swofford 2002). The test was run using 100 random stepwise additions and 1000 replicates of heuristic search with tree bisection–reconnection (TBR) branch swapping. The optimal partitioning strategy and evolutionary models for the combined sequence matrix were assessed with PartitionFinder (Lanfear et al. 2012) under the Bayesian Information Criterion (BIC) and using the implemented greedy algorithm.

Bayesian phylogenetic inference was conducted using MrBayes 3.2 (Ronquist et al. 2012). Two independent analyses consisting of four chains each were run for 5·10^6^ generations specifying a sampling frequency every 100 generations, and setting a burn-in fraction of 10%. MCMC convergence and the effective sample sizes (ESS) estimates were checked with TRACER v. 1.5 (Rambaut and Drummond 2007). Additionally, a maximum likelihood search was done using GARLI v.2.01 (Zwickl 2006) and performing 100 bootstrap replicates.

### Taxonomic hypotheses testing

Specific hypotheses of monophyly were tested using a ML framework and the Approximately Unbiased test (AU test, Shimodaira 2000) as implemented in the CONSEL program (Shimodaira and Hasegawa 2001). We compared our molecular phylogenetic hypothesis with some of the most relevant systematic proposals for the genus *Chrysolina* (see results). Prior to the evaluation of each taxonomic scenario, a ML phylogenetic analysis was performed in GARLI v.2.01 using the same partitioning scheme and models as in the phylogenetic searches described above, but enforcing the monophyly of the taxa of interest. Once the resulting ML trees were obtained, their per site log-likelihoods were calculated using RAxML v8.0.X program (Stamatakis 2014) and used as input data in CONSEL.

### Ancestral character reconstruction

Ancestral host plant affiliations were reconstructed using BayesTraits v. 2.0 (Pagel and Meade 2013) selecting the MCMC mode and the “multistate” model of evolution (Pagel et al. 2004). To take into account phylogenetic uncertainty, reconstructions were based on 1000 randomly selected post-burnin Bayesian trees from the phylogenetic analysis in MrBayes 3.2. Following the manual’s recommendations (http://www.evolution.rdg.ac.uk/BayesTraitsV2.0Files/TraitsV2Manual.pdf), the reversible-jump (RJ) MCMC with a hyperprior approach was chosen, and the interval of 0–30 for the RJ-hyperprior implementing an exponential distribution was applied. The “addMRCA” command was used to calculate the posterior distribution of ancestral character states at selected nodes in the Bayesian *Chrysolina* tree. A total of 10·10^6^ generations were run, with samples taken every 100 iterations and discarding a burn-in fraction of 10%. Results of the MCMC runs including the ESS values were analysed in TRACER v. 1.5.

We also used BayesTraits to evaluate different ancestral host plant affiliations scenarios at the root of the *Chrysolina* tree. Analyses were conducted by enforcing the ancestral state of the most recent common ancestor (mrca) for the core *Chrysolina* node (excluding the divergent species *Chrysolina vigintimaculata*) to be one of the eight host plant families recorded for the studied *Chrysolina* species. MCMC was used to explore the samples and the space rate parameter of 1000 post-burnin trees generated in the MrBayes analysis. We performed two independent runs of 10·10^6^ generations for each one of the constrained searches, and sampling rate parameters every 100 generations. The constrained runs were then compared by calculating the Bayes factors between the best and second best models based on the harmonic mean of the likelihood from each analysis as indicated in BayesTraits manual.

## Results

### Sequence data and phylogenetic analysis

Lengths of the amplified gene fragments ranged from 581 to 794 bp for *cox*1, 278 to 512 bp for *rrnL*, and 294 to 363 for H3. Total length of the concatenated DNA sequence matrix was 1682 bp. In *cox*1, 48.36% of the aligned positions were variable, indicating high divergence level among the studied sequences. Indeed, accumulation of mutations for *cox*1 was higher than for the other markers, as shown by the pairwise sequence divergence (*p*-distance), which ranged between 0.0063 and 0.2236 (average: 0.1331±0.0105) for *cox*1, 0.0012 and 0.1723 (average: 0.0924±0.0100) for *rrnL*, and 0.0027 and 0.1077 (average: 0.0641±0.0108) for H3. Also, *cox*1 and *rrnL* sequences showed the well-known A+T bias typical of insect mtDNA (69.9% and 76,4%, respectively), whereas base frequency was more balanced in the nuclear H3 marker (54,8%). Chi-squared tests for bias in base composition showed no significant heterogeneity in our datasets (P>0.99). On the other hand, ILD test revealed no evidence of incongruence among molecular markers (*P*= 0.24), and we therefore performed all subsequent phylogenetic analyses following a supermatrix approach.

**Figure 1.**
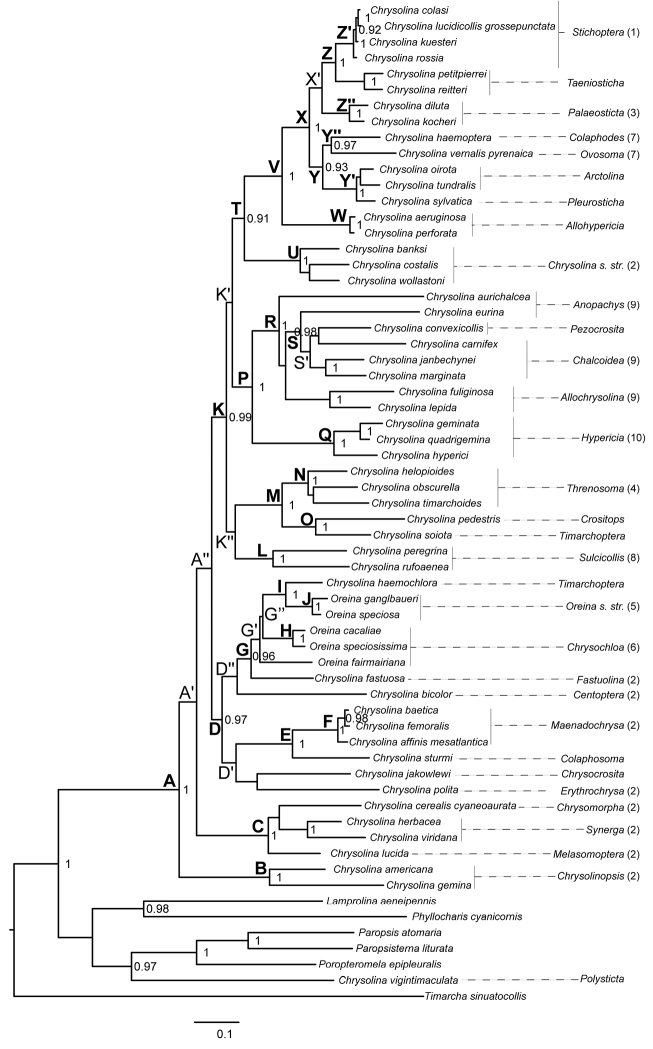
Bayesian phylogenetic tree obtained from the combined analysis of *cox*1, *rrn*L and H3. Node numbers represent Bayesian posterior probability values. Only support values higher than 0.9 are shown. Numbers accompanying the subgeneric classification of the *Chrysolina* species on the right correspond to the systematic groups defined by Bourdonné and Doguet (1991). Clades mentioned in the text are highlighted.

**Figure 2.**
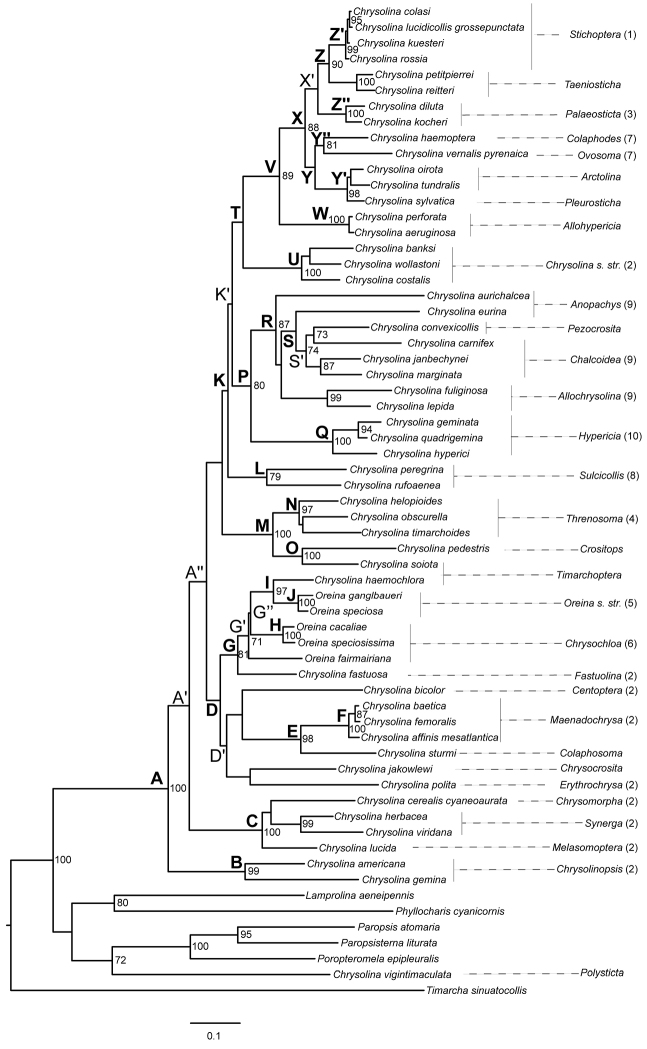
Maximum likelihood phylogenetic tree obtained from the combined analysis of *cox*1, *rrn*L and H3. Node numbers represent bootstrap support values. Only support values higher than 0.7 are shown. Numbers accompanying the subgeneric classification of the *Chrysolina* species on the right correspond to the systematic groups defined by Bourdonné and Doguet (1991). Clades mentioned in the text are highlighted.

The best-fit partitioning scheme selected by PartitionFinder under BIC divided the data into seven subsets, each with its own model of molecular evolution (Table 2). The effective sample size value for each parameter sampled from the MCMC analysis was always >200. Bayesian and ML searches resulted in almost the same topology (Figures 1 and 2), with few discrepancies affecting only unsupported relationships such as the placement of the species *Chrysolina bicolor* (Fabricius, 1775), the position of the subgenus *Sulcicollis* (Fairmaire, 1887), and the internal branching pattern of the three species of the subgenus *Chrysolina* *s. str.* Motschulsky. Both phylogenetic approaches also yielded similar results in terms of nodal support, differing mainly in the values associated to some of the basal nodes of the core *Chrysolina* clade, which were higher in the Bayesian analysis (e.g. nodes K, D and T). The resulting phylogenetic trees revealed the paraphyly of the genus *Chrysolina* as currently described, due to the inclusion of the *Oreina* representatives within the *Chrysolina* clade (Figures 1 and 2). The genus *Oreina* is also recovered as a paraphyletic clade that includes the species *Chrysolina haemochlora* (Gebler, 1823). The results showed the monophyly of the studied *Chrysolina* (plus *Oreina*) species [clade A, Bayesian posterior probability (*pp*)=1, bootstrap=100] excepting the African taxa Chrysolina (Polysticta) vigintimaculata, which showed a higher affinity with outgroup taxa. In addition, the monophyletic status of the subgenera with more than one species sampled in the study was recovered in all cases excepting *Anopachys* Motschulsky, *Chalcoidea*, *Timarchoptera* Motschulsky and *Oreina* subgenus *Chrysochloa* Hope. The inferred topology allowed for the identification of four main monophyletic subgenera assemblages within the core *Chrysolina* clade with high support values in at least one of the resulting trees (clades B, C, D and K). Within these main lineages, it was also possible to identify systematic relationships among subgenera at different phylogenetic levels. The inferred groups of phylogenetically related subgenera and their statistical supports are summarized in Table 3.

**Table 2.** Optimal partitioning strategy and evolutionary models selected using PartitionFinder under the Bayesian Information Criterion.

Partition	Model
*cox*1 codon pos. 1	GTR+I+G
*cox*1 codon pos. 2	HKY+I+G
*cox*1 codon pos. 3	GTR+G
*rrnL*	GTR+I+G
H3 codon pos. 1	SYM+G
H3 codon pos. 2	JC
H3 codon pos. 3	HKY+I+G

**Table 3.** Inferred phylogenetic relationships among *Chrysolina* and *Oreina* subgenera and their statistical supports. Nodes have been coded according to Figures 1 and 2.

Node (Bayesian posterior probability; ML bootstrap)						Subgenera included
B (1.00; 99)						*Chrysolinopsis*
C (1.00; 100)						*Chrysomorpha*
						*Melasomoptera*
						*Synerga*
D (0.97; <70)						*Centoptera*
						*Chrysocrosita*
						*Erythrochrysa*
	E (1.00; 98)					*Colaphosoma*
						*Maenadochrysa*
	G (0.96; 81)					*Fastuolina*
						*Oreina* subgenus *Chrysochloa*
		I (1.00; 97)				*Oreina* *s. str.*
						*Timarchoptera* partim.
K (0.99; <70)						*Sulcicollis*
	M (1.00; 100)					*Threnosoma*
		O (1.00; 100)				*Crositops*
						*Timarchoptera* partim.
	P (1.00; 80)					*Hypericia*
		R (1.00; 87)				*Anopachys*
						*Allochrysolina*
			S’ (<0.9; 74)			*Chalcoidea*
						*Pezocrosita*
	T (0.91; <70)					*Chrysolina* *s. str.*
		V (1.00; 89)				*Allohypericia*
			X (1.00; 88)			*Palaeosticta*
				Y (0.93; <70)	Y’ (1.00; 98)	*Arctolina*
						*Pleurosticha*
					Y’’ (0.97; 81)	*Colaphodes*
						*Ovosoma*
				Z (1.00; 90)		*Stichoptera*
						*Taeniosticha*

### Testing for monophyly of key groups

Constrained ML searches were used to evaluate a number of taxonomic hypotheses for *Chrysolina* and *Oreina* using the AU test (Table 4). The phylogenetic scenarios that were rejected in the analyses included the systematic placement of *Oreina* as a different genus from *Chrysolina* (P=0.016), the synonymy of subgenera *Paraheliostola* L. N. Medvedev and *Timarchoptera* (Mikhailov 2002, P=0.001), the suggestion of a close relationship between *Threnosoma* Motschulsky and Chrysolina (Timarchoptera) haemochlora (Mikhailov 2005, P<0.001), the reciprocal monophyly of *Colaphodes* and *Taeniochrysa* (Hsiao and Pasteels 1999, P<0.001), the inclusion of *Chrysolina timarchoides* (Brisout, 1882) within the subgenera *Maenadochrysa* Bechyné (Bieńkowski 2001, P<0.001), the recognition of *Craspeda* sensu Bourdonné 2005 as a different genus from *Chrysolina* (P<0.01), the segregation from *Chrysolina* of the subgenera *Allochrysolina* Bechyné, *Chalcoidea* and *Pezocrosita* Jakobson (Bourdonné 2012, P<0.01), the monophyly of the *Chrysolina* species belonging to the “group 2” described by Bourdonné and Doguet (1991) (P<0.001) (Table 1), as well as the monophyly of the *Chrysolina* species feeding on hosts from the same plant family (Apiaceae, Asteraceae, Lamiaceae, Plantaginaceae, Ranunculaceae and Scrophulariaceae; P≤0.001 in all cases). Conversely, the molecular data could not reject the reciprocal monophyly of several taxa assemblages, such as *Chrysolina vigintimaculata* and the rest of the studied *Chrysolina* species (P=0.165), *Chrysolina* species belonging to the “group 6” described by Bourdonné and Doguet (1991) (P=0.527) (Table 1), subgenera *Allochrysolina* and *Anopachys* (Hsiao and Pasteels 1999, P=0.215), subgenera *Chalcoidea* and *Hypericia* (Pasteels et al. 2003, P=0.066), subgenera *Allochrysolina*, *Chalcoidea* and *Pezocrosita* (Bourdonné 2012, P=0.205), and the subgenera *Palaeosticta* Bechyné and *Taeniosticha* Motschulsky (Bourdonné 2005, P=0.198). Also, the monophyly of the sampled species concerning the subgenera *Anopachys*, *Chalcoidea* and *Oreina* subgenus *Chrysochloa* could not be rejected (P≥0.212 in all cases).

**Table 4.** Results of the Approximately Unbiased test (AU test, Shimodaira 2000). Statistically significant P values are indicated in bold (P < 0.05).

Hypothesis of monophyly	Authorship	AU test
*Chrysolina timarchoides* + *Maenadochrysa*	Bienkowski (2001)	**0.000**
*Palaeosticta* + *Taeniosticha*	Bourdonné (2005)	0.198
*Craspeda* as a different genus from *Chrysolina*	Bourdonné (2005)	**0.007**
*Allochrysolina* + *Chalcoidea* + *Pezocrosita*	Bourdonné (2012)	0.205
*Allochyrsolina* + *Chalcoidea* + *Pezocrosita* as a different genus from *Chrysolina*	Bourdonné (2012)	**0.003**
Species “group 2”	Bourdonné and Doguet (1991)	**0.000**
Species “group 6”	Bourdonné and Doguet (1991)	0.527
*Allochrysolina* + *Anopachys*	Hsiao and Pasteels (1999)	0.215
*Colaphodes* + *Taeniochrysa*	Hsiao and Pasteels (1999)	**0.000**
*Paraheliostola* + *Timarchoptera*	Mikhailov (2002)	**0.001**
*Chrysolina haemochlora* + *Threnosoma*	Mikhailov (2005)	**0.000**
*Chalcoidea* + *Hypericia*	Pasteels et al. (2003)	0.066
*Anopachys* species		0.212
*Chalcoidea* species		0.383
*Chrysochloa* species		0.528
*Oreina* as a different genus from *Chrysolina*		**0.016**
*Chrysolina vigintimaculata* + rest of the *Chrysolina* species + *Oreina*		0.165
Species feeding on Apiaceae		**0.000**
Species feeding on Asteraceae		**0.000**
Species feeding on Lamiaceae		**0.000**
Species feeding on Plantaginaceae		**0.000**
Species feeding on Ranunculaceae		**0.001**
Species feeding on Scrophulariaceae		**0.000**

### Ancestral character reconstruction

**Figure 3.**
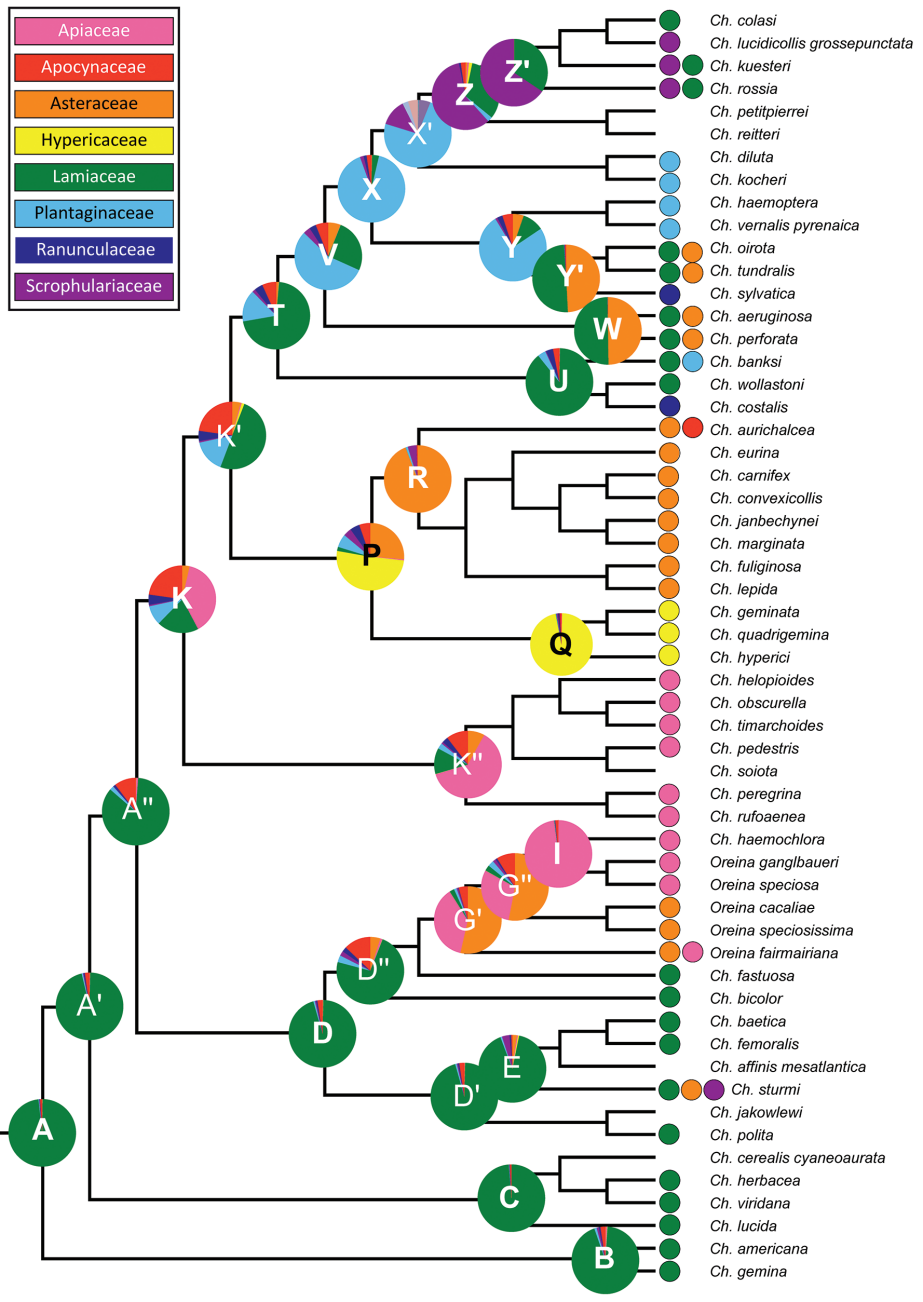
Ancestral reconstruction of host plant affiliations in the studied species of *Chrysolina* and *Oreina*. Terminal taxa are coded according to the available host plants records from the literature (Table 1). Pie charts at selected nodes show probabilities of each state from the Bayesian analysis in BayesTraits. Clades mentioned in the text are highlighted.

The Bayesian reconstruction of ancestral host plant associations showed an ancient affiliation with Lamiaceae at the root of the core *Chrysolina* clade (Figure 3, node A, P=0.98; Table 5). This plant family was also recovered as the most likely ancestral host for three of the main clades in our molecular phylogeny (nodes B, C and D; P=0.94, 0.99 and 0.95, respectively). Within clade D, a host shift from Lamiaceae towards Asteraceae (P=0.54) and/or Apiaceae (P=0.37) was detected for the mrca of *Oreina* and Chrysolina (Timarchoptera) haemochlora (clade G’). On the other hand, ancestral host plant reconstruction for node K was ambiguous, recovering associations with multiple families. However, it was possible to identify the occurrence of several host shifts for its derived lineages towards a new trophic association with (i) Apiaceae (node K’’, P=0.62), (ii) Hypericaceae (nodes P and Q, P=0.51 and 0.97, respectively), (iii) Asteraceae (node R, P=0.94), (iv) Plantaginaceae (node X, P=0.91), and (v) Scrophulariaceae (node Z’, PP=0.66). Nodes W and Y’ respectively showed a reversal shift from an ancestral Plantaginaceae host to the original Lamiaceae host family (P=0.5) as well as a new trophic link with Asteraceae (P=0.5).

**Table 5.** Posterior probability values of ancestral host-plant affiliations calculated in BayesTraits for the selected nodes in the *Chrysolina*-*Oreina* phylogeny. The highest probability value(s) for each node are highlighted in bold. Ast.=Asteraceae, Api.=Apiaceae, Hyp.=Hypericaceae, Lam.=Lamiaceae, Plant.=Plantaginaceae, Scro.=Scrophulariaceae, Ran.=Ranunculaceae, Apo.=Apocynaceae.

	Host-plant family							
Node	Ast.	Api.	Hyp.	Lam.	Plant.	Scro.	Ran.	Apo.
A	0.000	0.001	0.000	**0.980**	0.003	0.002	0.002	0.010
A’	0.001	0.002	0.001	**0.959**	0.006	0.003	0.006	0.022
A’’	0.002	0.010	0.000	**0.852**	0.020	0.001	0.011	0.104
B	0.002	0.006	0.002	**0.937**	0.011	0.010	0.008	0.024
C	0.000	0.000	0.000	**0.987**	0.002	0.002	0.001	0.007
D	0.002	0.001	0.000	**0.952**	0.008	0.006	0.006	0.024
D’	0.002	0.001	0.000	**0.952**	0.008	0.006	0.006	0.024
D’’	0.048	0.010	0.001	**0.732**	0.033	0.023	0.024	0.129
E	0.022	0.005	0.006	**0.910**	0.008	0.036	0.008	0.006
G’	**0.536**	0.374	0.001	0.023	0.012	0.008	0.002	0.044
G’’	**0.531**	0.300	0.001	0.027	0.029	0.015	0.009	0.089
I	0.001	**0.979**	0.000	0.001	0.001	0.000	0.002	0.015
K	0.036	**0.387**	0.000	0.200	0.093	0.007	0.049	0.227
K’	0.040	0.005	0.013	**0.499**	0.158	0.007	0.049	0.227
K’’	0.080	**0.624**	0.001	0.124	0.028	0.009	0.029	0.104
P	0.262	0.005	**0.511**	0.019	0.064	0.039	0.047	0.053
Q	0.001	0.002	**0.967**	0.001	0.003	0.008	0.008	0.010
R	**0.941**	0.000	0.000	0.000	0.010	0.042	0.001	0.006
T	0.011	0.001	0.001	**0.709**	0.153	0.015	0.041	0.068
U	0.001	0.001	0.001	**0.890**	0.039	0.004	0.034	0.031
V	0.059	0.001	0.001	0.257	**0.555**	0.034	0.033	0.060
W	**0.498**	0.000	0.000	**0.501**	0.000	0.001	0.000	0.001
X	0.003	0.000	0.000	0.033	**0.908**	0.018	0.014	0.023
X’	0.005	0.000	0.001	0.055	**0.736**	0.128	0.028	0.047
Y	0.052	0.000	0.000	0.103	**0.757**	0.011	0.026	0.050
Y’	**0.492**	0.000	0.000	**0.498**	0.001	0.001	0.002	0.006
Z	0.009	0.008	0.016	0.327	0.023	**0.586**	0.009	0.023
Z’	0.000	0.000	0.000	0.344	0.000	**0.656**	0.000	0.000

Results from Bayes factor comparisons of the constraint hypotheses for the ancestral plant family at the root of the core *Chrysolina* clade (node A) corroborated MCMC ancestral state reconstruction, offering positive to very strong statistical support for an ancestral trophic association with Lamiaceae (Table 6).

**Table 6.** Comparing model support with the Bayes factor. Bayes factors were calculated as described in the BayesTraits manual: BF=2(ln LhA−ln LhB), where ln Lhx is the marginal likelihood from the harmonic mean of the post-convergence. The plant family Lamiaceae is the most likely ancestral host at the root of the core *Chrysolina* clade with the highest harmonic mean. The right column indicates the Bayes factor compared against Lamiaceae as the favoured ancestral host. * Indicates positive evidence, ** indicates strong evidence, and *** indicates very strong evidence for the favoured hypothesis.

Host plant family	ln Lh	Bayes Factor
Apiaceae	-62.77	5.27**
Apocynaceae	-63.78	7.30**
Asteraceae	-65.71	11.16***
Hypericaceae	-65.59	10.92***
Lamiaceae	-60.13	-
Plantaginaceae	-62.44	4.61*
Ranunculaceae	-62.57	4.86*
Scrophulariaceae	-63.24	6.20**

## Discussion

### Molecular systematics of *Chrysolina*

The mitochondrial and nuclear genes used here provided an expanded and better-resolved tree topology for the genus *Chrysolina*, significantly improving previous phylogenetic hypotheses. Our results support the reciprocal monophyly of the studied species of *Chrysolina* (plus *Oreina*) including the divergent Chrysolina (Polysticta) vigintimaculata, whose relationship with the core *Chrysolina*-*Oreina* clade could not be rejected by the AU test. The inferred tree topologies recovered *Chrysolina vigintimaculata* as a well-differentiated lineage sister to the rest of the ingroup taxa. This species has been traditionally assigned to the subgenus *Atechna* Chevrolat (Bieńkowski 2001), a species of which was included in the phylogenetic analysis of Gómez-Zurita et al. (2008) based on three ribosomal genes and showing a clear divergence from the *Chrysolina*-*Oreina* clade. In addition, the same pattern was observed in a different phylogenetic study based on five molecular markers (Jurado-Rivera et al. in prep.) that included the species Chrysolina (Atechna) striata (Degeer, 1778). Although more data are needed, the available information indicates that these taxa may represent a lineage of early divergence within *Chrysolina* whose taxonomic status should be further investigated.

The inferred topology also supported most of the current subgeneric taxonomy of *Chrysolina* (Bieńkowski 2001, Kippenberg 2010), since the monophyly of the subgenera screened for more than one species could be demonstrated or alternatively could not be rejected by the AU test. The exceptions in this regard are the synonymy of the subgenus *Paraheliostola* with the subgenus *Timarchoptera* by Mikhailov (2002) and the combination of the species Chrysolina (Threnosoma) timarchoides with the subgenus *Maenadochrysa* by Bieńkowski (2001). In both cases the taxa in question were recovered with support as well-differentiated lineages, thus indicating that such taxonomic decisions could be wrong. Therefore, the subgenus *Paraheliostola* (type species *Chrysolina soiota* Jacobson, 1924) should be restored according to the present molecular phylogeny. Moreover, the available karyological evidence also conflicts with Bieńkowski’s (2001) proposal (Petitpierre 1975, 1981), and we thus agree with Daccordi and Ruffo (2005) and with Kippenberg (2010) in that *Chrysolina timarchoides* belongs in the subgenus *Threnosoma*.

The new molecular phylogeny also sheds light on the contentious issue of the taxonomic status of *Oreina*. Our analyses supported the inclusion of the studied *Oreina* species within the core *Chrysolina* clade, which was also backed up statistically in the AU test constraining these genera to be reciprocally monophyletic (Table 4). The sample included the type species of the genus, *Oreina speciosa* (Linnaeus, 1758), which further strengthens our findings and corroborates previous hypotheses that consider *Oreina* as part of the *Chrysolina* lineage (Chapuis 1874, Bourdonné and Doguet 1991, Daccordi 1994). Moreover, the species feeding on Apiaceae hosts, *Oreina ganglbaueri* (Jakob, 1953) and *Oreina speciosa*, were recovered as more closely related to the also Apiaceae feeding *Chrysolina haemochlora* than to the remainder of the *Oreina* species analysed here, reinforcing our conclusions and highlighting the need for a taxonomic revision for the group. On the other hand, the proposal of considering the genera *Craspeda* and *Chalcoidea* (*sensu* Bourdonné 2005 and 2012, respectively) as separate lineages from the remainder of the *Chrysolina* species is not supported in our phylogenetic framework, although the monophyly of the taxa included in each of them could not be statistically rejected (Table 4). Thus, the recognition of *Craspeda* and/or *Chalcoidea* as valid genera would render *Chrysolina* paraphyletic.

Excluding the divergent species *Chrysolina vigintimaculata*, *Chrysolina* could be subdivided into four major clades (Figures 1 and 2, clades B, C, D and K). The clades B and C comprised species from the “group 2” defined by Bourdonné and Doguet (1991), all of them feeding on host plants belonging to the family Lamiaceae and with a diploid chromosome number of 2n=24 (Petitpierre 1975, 1981, 1983). The hypothetical monophyly of the aforementioned “group 2” was statistically rejected by the AU test, thus reinforcing our finding that such an assemblage of species does not constitute a natural group. The clade B included two monotypic subgenera (*Chrysolinopsis* Bechyné and *Taeniochrysea*, *sensu* Bieńkowski 2001) that have been recently regarded as synonyms by Kippengberg (2010), a taxonomic decision that is strongly supported in our phylogenetic analyses. The monophyly of the species nested in clade C were also noted in the phylogenetic study of Garin et al. (1999), excepting the species *Chrysolina cerealis* (Linnaeus, 1767) that they recovered in a divergent clade as sister to *Chrysolina fastuosa* with maximum bootstrap support. Here we have analysed the subspecies *Chrysolina cerealis cyaneoaurata* (Motschulsky, 1860) inferring a clear relationship with the remainder of the members in clade C that is supported with maximum posterior probability and bootstrap values. Genetic distances (*p*-distance) between the sequences deposited in GenBank by Garin et al. (1999) regarding *Chrysolina cerealis* and our data for *Chrysolina cerealis cyaneoaurata* were unusually high for an intraspecific comparison (*cox*1: 0.14; *rrnL*: 0.08), thus suggesting that the taxa in question do not belong to the same species. It remains to be investigated whether their divergence is due to specimen misidentification or whether *Chrysolina cerealis* *s. str.* and *Chrysolina cerealis cyaneoaurata* really are different species. Meanwhile, the results about the systematic position of *Chrysolina cerealis* should be interpreted with caution.

Clade D defined the monophyletic origin of seven *Chrysolina* subgenera traditionally associated with the “group 2” proposed by Bourdonné and Doguet (1991) plus two *Oreina* subgenera included in “groups 5 and 6”, all of them with a karyotype 2n = 24 (Petitpierre 1975, 1981, 1983) excepting *Chrysolina haemochlora* (2n=27, Petitpierre and Mikhailov 2009). The affinity between the subgenera *Colaphosoma* Motschulsky and *Maenadochrysa* could be established with confidence agreeing with their shared feeding habits on Lamiaceae species of the tribe Mentheae (Jolivet and Petitpierre 1976, Jolivet et al. 1986, Bieńkowski 2010). On the other hand, the close relationship recovered in the present work among *Chrysolina fastuosa* and the studied *Oreina* species is consistent with the findings of Hsiao and Pasteels (1991) based on a different set of molecular markers. The authors concluded that such association was contradicted by strong morphological evidence, highlighting the need of further research on this issue. Our molecular phylogeny not only confirmed the monophyly of these taxa, but also revealed the inclusion of an additional *Chrysolina* species in this clade, *Chrysolina haemochlora*.

Interestingly, our results regarding the clade K were fully consistent with most species groupings established by Bourdonné and Doguet (1991) based on morphology, karyology and biology of the species (“groups 1, 3, 4, 7, 8, 9, 10 and 2 partim.”). Available molecular phylogenies of *Chrysolina* (Garin et al. 1999, Hsiao and Pasteels 1999) failed at recovering supported relationships among these groups, excepting the monophyletic origin of the species belonging in the “groups 1, 3 and 7” inferred by Garin et al. (1999). In contrast, our analyses allowed for the identification of their phylogenetic relationships at deep taxonomic level, and also extended the results to seven *Chrysolina* subgenera not studied by Bourdonné and Doguet (1991). The latter was the case of clade M, where the subgenera *Crositops* Marseul and *Timarchoptera* (more likely *Paraheliostola*, see above) were recovered as the sister lineage of the *Threnosoma* species regarded as “group 4”. Indeed, the subgenera *Crositops* and *Threnosoma* are known to share morphological attributes (Mikhailov 2005). Although no information is available for the species *Chrysolina soiota*, the remainder of the species in clade M feed on Apiaceae and also share a male karyotype 2n=47 (Petitipierre 1981, 1999, Petitpierre et al. 2004, Petitpierre and Mikhailov 2009), which is highly consistent with their close association recovered here. On the other hand, the existence of a relationship between the Mediterranean subgenus *Threnosoma* and the Siberian subgenus *Timarchoptera* proposed by Mikhailov (2005) was rejected by the AU test. Another subgenus that was not analysed by Bourdoneé and Doguet (1991) is represented in our sampling by the species Chrysolina (Pezocrosita) convexicollis (Jakobson, 1901), which appeared in the trees clearly nested within the species “group 9” (clade R) sharing with them a trophic link with Asteraceae. Our phylogenetic hypotheses also allowed for the identification of two main evolutionary lineages within “group 9”, on one hand the species belonging in the subgenera *Anopachys* [excluding *Chrysolina aurichalcea* (Gebler, 1825)], *Chalcoidea* and *Pezocrosita*, all of them feeding on closely related plant species in the family Asteraceae in the tribe *Anthemideae* (*Achillea*, *Artemisia*, *Santolina*, *Tanacetum*; Cobos 1953, Jolivet and Petitpierre 1976, Bieńkowski 2010, 2011, clade S) and sharing a karyotype of 2n=40 [cytogenetic data for *Chrysolina eurina* (Frivaldszky, 1883) and *Chrysolina convexicollis* are not available], and on the other hand the species in the subgenera *Allochrysolina* with a male karyotype 2n=42 (Petitpierre 1999) and feeding on closely related Asteraceae host plants in the subtribe Centaureinae (*Centaurea*, *Mantisalca*, Jolivet and Petitpierre 1976, Bourdonné and Doguet 1991). In turn, the species in “group 9” were recovered as the sister lineage of the species classified in the “group 10” (subgenus *Hypericia*; clade Q), thus contradicting Bourdonné and Doguet’s (1991) view that the subgenus *Hypericia* is so differentiated from the remainder of the *Chrysolina* subgenera that it deserves a generic status. Recognition of the genus *Hypericia* would render *Chrysolina* paraphyletic. Also regarding this lineage, Pasteels et al. (2003) found that the subgenera *Hypericia*, *Chalcoidea* and *Sphaeromela* are the only Chrysomelinae leaf beetles producing polyoxygenated steroids as defensive toxins, and suggested that they could be raised to a distinct genus. However, our inferred topologies were not compatible with this hypothesis, although the AU test could not reject the constrained monophyly of *Chalcoidea* and *Hypericia*. On the other hand, the well-supported and resolved clade T allowed for the identification of the phylogenetic relationships among four of the systematics groups defined by Bourdonné and Doguet (1991), and also expanded our knowledge regarding the systematic position of four subgenera not included before in any phylogenetic analysis. The species in the subgenera *Chrysolina* *s. str.* were placed in the “group 2” based on their trophic link with the plant family Lamiaceae but our results clearly contradict this association (clade U), agreeing with their unique male karyotype (2n=23; Petitpierre 1975, 1981, 1983). The common ancestry of *Colaphodes*, *Ovosoma* Motschulsky, *Palaeosticta* and *Stichoptera* Motschulsky demonstrated by Garin et al. (1999) was confirmed here, and in addition we show that the subgenera *Allohypericia* Bechyné, *Arctolina* Kontkanen, *Pleurosticha* Motschulsky and *Taeniosticha* also belong in this monophyletic lineage. The close relationship between the subgenera *Arctolina* and *Pleurosticha* has been previously proposed according to their morphology (Bieńkowski 2004) and their karyological resemblances [2n=26 (Xy_p_), Petitpierre and Mikhailov 2009]. In this regard, our study contributes additional evidence confirming their phylogenetic relatedness (clade Y’). The monophyly of the species adapted to the plant family Plantaginaceae (subgenera *Palaeosticta*, *Colaphodes* and *Ovosoma*) could not be rejected, indicating that they could conform to a natural group, thus expanding Bourdonné and Doguet’s (1991) “group 7”. On the other hand, the *Stichoptera* species of the “group 1” *sensu* Bourdonné and Doguet (1991) were demonstrated to be sister to the morphologically well-defined subgenus *Taeniosticha* (Bourdonné et al. 2013). *Stichoptera* species are characterized by their marked asymmetrical karyotypes (Petitpierre 1999) and their affiliation with Lamiaceae and Scrophulariaceae host plants, but unfortunately no data are available regarding the biology and the cytogenetics of the subgenus *Taeniosticha* to contrast with our molecular results.

### Evolution of the host plant associations in *Chrysolina*

The initial stages of the evolutionary history of the genus *Chrysolina* were closely related to the plant family Lamiaceae (Figure 3, node A), which is in line with the pioneering studies based on the karyology and the ecology of the species (Petitpierre and Segarra 1985, Bourdonné and Doguet 1991) and also on mtDNA sequences (Garin et al. 1999). The inferred ancestral association with Lamiaceae was highly favoured in our analyses compared to the alternative hypotheses, including an original affiliation with the family Asteraceae suggested by Crowson (1981).

The most basal clades in our *Chrysolina* phylogeny are those living on Lamiaceae. However, the phylogenetic uncertainty affecting this region of the tree prevents us for drawing firm conclusions about the number of lineages that have adapted to this plant family at the early stages of the evolution of the genus. In contrast, our phylogenetic analyses allowed for the identification of a minimum of eight host plant family shifts in the *Chrysolina* tree, thus indicating that the feeding spectrum of the extant *Chrysolina* species is the result of frequent and abrupt host shifts in their evolutionary history. While some of these shifts are between plant families belonging to the same order (Lamiaceae, Plantaginaceae, Scrophulariaceae; order Lamiales; APG 2009), others are between distant plant families from different subclasses [shift from families in the subclass Asterids to Hypericaceae (subclass Rosids); APG 2009] or even from more divergent lineages [shifts from Asterids to Ranunculaceae (basal Eudicot); APG 2009]. Three main hypotheses have been proposed concerning the macroevolution of insect–plant associations (Nyman 2010): (i) the ‘cospeciation’ or ‘parallel cladogenesis’ model (Fahrenholz 1913): matching of speciation events between insects and their host plants; (ii) the ‘escape and radiate’ model (Ehrlich and Raven 1964): plants ‘escape‘ from herbivory due to novel defences and radiate, followed by colonization of new insect taxa that then radiate on them; and (iii) the ‘sequential evolution’ model (Jermy 1984): insects have little effect on the speciation of their hosts, whereas the diversification of hosts increases possibilities of ecological speciation in insects. The hypothesis of ‘parallel cladogenesis’ between *Chrysolina* lineages and their host plant families can be discarded as the temporal origin of the more closely related host plant families recorded for *Chrysolina* (Lamiaceae and Scrophulariaceae: mrca >65Ma, Bremer et al. 2004) clearly pre-dates the diversification of the *Chrysolina* lineage itself [mrca *ca.* 40Ma, (*ca.* 20Ma excluding the divergent subgenera *Atechna*), Gómez-Zurita et al. 2007]. Consistently, this pattern of asynchronous diversification has been found among other phytophagous insect groups and their host plants (Lopez-Vaamonde et al. 2006, McKenna et al. 2009). Regarding the ‘escape and radiate’ model, the existence of coincident radiations at a large scale among host families and the *Chrysolina* lineages is also not possible due to this time lag in their respective origins. Conversely, the ancestral host plant family affiliations inferred for *Chrysolina* seem to fit better the ‘sequential evolution’ model, as deduced from the continuous host-shifting among pre-existing host families that characterizes the evolution of the genus (Nyman 2010). Indeed, some *Chrysolina* clades have experienced multiple host shifts from the ancestral affiliation with Lamiaceae. As an example we could cite the case of the preference for Lamiaceae observed in the derived lineages *Allohypericia* (clade W), *Stichoptera* (clade Z’), *Arctolina* and *Pleurosticha* (clade Y’), which seems to be a back-colonization of this family from ancestors previously adapted to Plantaginaceae. Another case of multiple shifts is illustrated by the transition from Lamiaceae to Asteraceae and then to Apiaceae inferred for the *Oreina* clade, which is highly consistent with previous results based on allozyme data (Dobler et al. 1996) and mtDNA sequences (Hsiao and Pasteels 1999). In addition, convergent shifts to the same host plant family in different *Chrysolina* lineages have also occurred (Apiaceae: clades G’ and K’’; Asteraceae: clades G’, R, W’ and Y’, *Chrysolina sturmi* (Westhoff, 1882) and *Chrysolina cerealis cyaneoaurata*; Ranunculaceae: *Chrysolina costalis* (Olivier, 1807) and *Chrysolina silvatica* (Gebler, 1823); Scrophulariaceae: clade Z’ and *Chrysolina sturmi*), thus suggesting the existence of evolutionary constraints in host shifts as it has been described in other phytophagous insects including Chrysomelidae (Futuyma et al. 1993, Futuyma and Mitter 1996, Janz et al. 2001, Nosil 2002). A possible explanation for the continuous and convergent shifts among restricted sets of plant taxa is the phytochemical similarity among the alternative hosts (Feeny 1992), and indeed this seems to be the underlying mechanism in other herbivorous beetle groups (Becerra 1997, Kergoat et al. 2005). It also has been suggested that convergent shifts may not be independent, in the sense that an ancestral trait allowing the colonisation of a given plant group might have been already present in the insect lineages (Janz and Nylin 2008).

*Chrysolina* leaf beetles are highly specialized herbivores feeding on a narrow range of host plants (Jolivet and Petitpierre 1976, Bourdonné and Doguet 1991). However, despite the high level of specialization, their diet breadth ranges from species feeding on few plant species from the same genus or family (*i.e.*, monophagous or oligophagous, respectively) to more generalist species exploiting few species but from different plant families (*i.e.*, polyphagous). In this regard, Garin et al. (1999) reported the subgenus *Chrysolina* *s. str.* as the only lineage within the genus experiencing a shift to a generalist feeding habit at the plant family level. Now, our expanded taxon sampling coupled with the availability of a more complete host plant record shows that polyphagy is distributed across the *Chrysolina* tree, although it occurs at a lower frequency than mono- and oligophagy. Moreover, our results suggest that niche widths have varied through time, since some *Chrysolina* clades include mixtures of species with different levels of diet breadth (clades E, G’, R, U, Y’ and Z’). Oscillations in host range over evolutionary time are thought to play an important role in the diversification of the phytophagous insects (oscillation hypothesis, Janz et al. 2006, Janz and Nylin 2008). Under this model, speciation is driven by successive cycles of expansion of the host-plant range and generation of new species through specialization on different hosts. The oscillations are maintained through the ability to retain essential parts of the genetic “machinery” to utilize ancestral hosts, and therefore the probability of a major host shift seems to be positively inﬂuenced by polyphagy (Janz 2011). Our results on *Chrysolina* are still too preliminary to offer any scenario for the evaluation of this hypothesis. However, as it has been shown here, the evolutionary history of the genus is deeply associated with the occurrence of frequent and abrupt host shifts giving rise to the specialization on a restricted set of divergent host plant taxa, which is consistent with the model predictions. Optimizing niche width on the *Chrysolina* phylogeny would help in elucidating whether the diet breadth of the extant polyphagous species indeed represent an event of host range expansion from specialized ancestors, and whether polyphagy has been a transitional stage during host shifts. However, ancestral host range reconstruction will require very detailed information on host plant records and a well-resolved phylogeny for all *Chrysolina* species (Janz and Nylin 2008). In this respect, future research will be directed towards the expansion of the taxonomic sampling and the exploration of additional molecular markers in order to improve phylogenetic resolution. The implementation of DNA-based techniques for the taxonomic identification of the host plants (Jurado-Rivera et al. 2009) would also contribute to our understanding on the evolution of the ecological associations in this large and highly diversified leaf-beetle genus.

## Conclusions

The combined phylogenetic analysis of mitochondrial (*cox*1 and *rrnL*) and nuclear (H3) DNA sequences allows for the identification of the main evolutionary lineages in a sample of *Chrysolina* species representing almost half of the subgeneric diversity and most of the morphological and ecological variation in the genus. Our results reveal the paraphyly of the genus *Chrysolina* as currently described, due to the inclusion of the *Oreina* representatives within the *Chrysolina* clade. In this regard, the recognition of the genera *Craspeda* and *Chalcoidea* (*sensu* Bourdonné 2005 and 2012, respectively) would also render *Chrysolina* paraphyletic. The molecular phylogeny support for the reciprocal monophyly of the studied species of *Chrysolina* (plus *Oreina*) including the divergent Chrysolina (Polysticta) vigintimaculata, whose relationship with the core *Chrysolina* clade cannot be statistically rejected. The molecular data are consistent with the current subgeneric arrangement of the species, excepting the synonymy of the subgenus Paraheliostola with the subgenus Timarchoptera by Mikhailov (2002) and the combination of the species Chrysolina (Threnosoma) timarchoides with the subgenus *Maenadochrysa* by Bieńkowski (2001). In addition, our hypothesized molecular phylogeny allows for the identification of deep-level evolutionary relationships among the studied *Chrysolina* subgenera. The Bayesian reconstruction of the host plant associations in the *Chrysolina* phylogeny points to the family Lamiaceae as the ancestral host of the genus, in agreement with previous studies. The feeding spectrum of the extant *Chrysolina* species has been shaped by continuous host-shifting among pre-existing host plant families throughout the evolution of the genus. Many clades include mixtures of species with different levels of diet breadth, indicating that niche width has varied through time.
